# Selective AKT kinase inhibitor capivasertib in combination with fulvestrant in *PTEN*-mutant ER-positive metastatic breast cancer

**DOI:** 10.1038/s41523-021-00251-7

**Published:** 2021-04-16

**Authors:** Lillian M. Smyth, Gerald Batist, Funda Meric-Bernstam, Peter Kabos, Iben Spanggaard, Ana Lluch, Komal Jhaveri, Andrea Varga, Andrea Wong, Alison M. Schram, Helen Ambrose, T. Hedley Carr, Elza C. de Bruin, Carolina Salinas-Souza, Andrew Foxley, Joana Hauser, Justin P. O. Lindemann, Rhiannon Maudsley, Robert McEwen, Michele Moschetta, Myria Nikolaou, Gaia Schiavon, Pedram Razavi, Udai Banerji, José Baselga, David M. Hyman, Sarat Chandarlapaty

**Affiliations:** 1 Memorial Sloan Kettering Cancer Center, New York, NY USA; 2 Segal Cancer Centre, Jewish General Hospital, McGill University, Montreal, QC Canada; 3 MD Anderson Cancer Center, Houston, TX USA; 4 University of Colorado Cancer Center, Aurora, CO USA; 5 Rigshospitalet, Copenhagen, Denmark; 6 Hospital Clínico Universitario de Valencia, University of Valencia, INCLIVA Biomedical Research Institute, Valencia, Spain; 7 Biomedical Research Centre Network in Cancer (CIBERONC), Madrid, Spain; 8 Institute Gustave Roussy, Villejuif, France; 9 National University Hospital, Singapore, Singapore; 10 Research and Early Development, Oncology R&D, AstraZeneca, Cambridge, UK; 11 Institute of Cancer Research and The Royal Marsden NHS Foundation Trust, London, UK; 12 Present Address: Loxo Oncology Inc., Stamford, CT USA; 13 Present Address: AstraZeneca, Gaithersburg, MD USA

**Keywords:** Breast cancer, Breast cancer

## Abstract

Five to ten percent of ER+ metastatic breast cancer (MBC) tumors harbor somatic *PTEN* mutations. Loss of function of this tumor-suppressor gene defines a highly aggressive, treatment-refractory disease for which new therapies are urgently needed. This Phase I multipart expansion study assessed oral capivasertib with fulvestrant in patients with *PTEN*-mutant ER+ MBC. Safety and tolerability were assessed by standard methods. Plasma and tumor were collected for NGS and immunohistochemistry analyses of PTEN protein expression. In 31 eligible patients (12 fulvestrant naive; 19 fulvestrant pretreated), the 24-week clinical benefit rate was 17% in fulvestrant-naive and 42% in fulvestrant-pretreated patients, with objective response rate of 8% and 21%, respectively. Non-functional *PTEN* was centrally confirmed in all cases by NGS or immunohistochemistry. Co­mutations occurred in *PIK3CA* (32%), with less *ESR1* (10% vs 72%) and more *TP53* (40% vs 28%) alterations in fulvestrant-naive versus fulvestrant-pretreated patients, respectively. *PTEN* was clonally dominant in most patients. Treatment-related grade ≥3 adverse events occurred in 32% of patients, most frequently diarrhea and maculopapular rash (both *n* = 2). In this clinical study, which selectively targeted the aggressive *PTEN*-mutant ER+ MBC, capivasertib plus fulvestrant was tolerable and clinically active. Phenotypic and genomic differences were apparent between fulvestrant-naive and -pretreated patients.

Trial registration number for the study is NCT01226316.

## Introduction

**Fig. 1:**
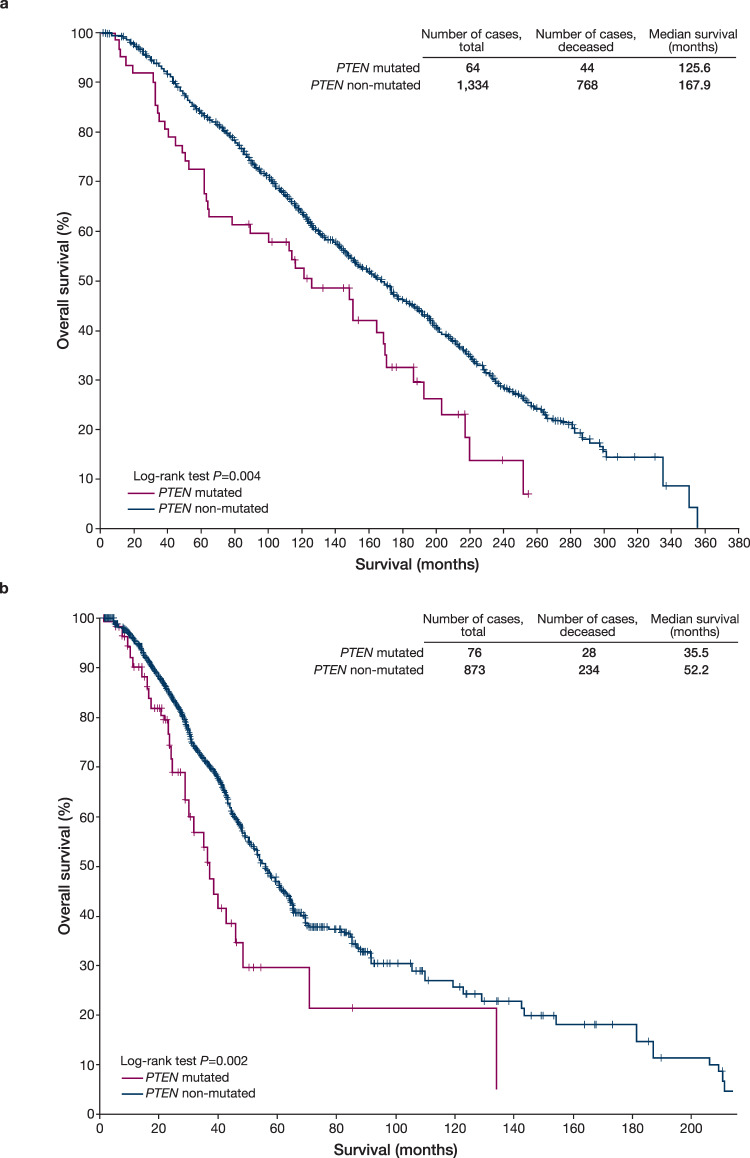
*PTEN* mutations are associated with a poor prognosis in ER+ HER2– breast cancer. **a** Early-stage ER+ HER2– breast cancers (METABRIC data)^19^. **b** Metastatic ER+ HER2– breast cancers (MSK-IMPACT data)^12^. Kaplan–Meier survival analysis for ER+ HER2– breast cancer patients by *PTEN* status in early-stage and metastatic BC: **a** overall survival of patients with ER+ HER2– primary breast tumors (*n* = 1398) by *PTEN* mutation status, using the same criteria employed in this study for enrollment; **b** overall survival from time of metastatic recurrence of patients with metastatic ER+ HER2– breast cancer (*n* = 949) by *PTEN* status. A patient with multiple metastatic samples sequenced by next-generation sequencing was considered *PTEN* altered if at least one sample harbored an eligible *PTEN* alteration. Overall survival for the METABRIC data utilized univariable or multivariable Cox proportional hazards models to examine the association between mutations and survival. Breast cancer-specific survival was used as the endpoint. Patients with deaths from other or unknown causes were censored at the date of death, and all other patients were censored at the date of last contact^19^. Overall survival for the MSK-IMPACT data, as defined by time of metastatic recurrence until death or last follow-up, was analyzed utilizing the MSK cohort^12^ restricted to patients with metastatic ER+/HER2– disease (*n* = 949 patients). Univariate *P* values were calculated using the log-rank test. The models were further adjusted using left truncation methods^37^ for late entry when tumor sequencing to assess *PTEN* status was performed after metastatic recurrence. ER+ estrogen-receptor-positive, HER2– human epidermal growth factor receptor negative.

Breast cancer (BC) is the most frequently diagnosed malignancy and the leading cause of cancer mortality in women^1^, with estrogen-receptor-positive (ER+), human epidermal growth factor receptor 2 negative (HER2–) BC being the most common BC subtype^2^. Within this heterogeneous subtype, 5–10% harbor somatic mutations in *PTEN*, a frequently mutated tumor-suppressor gene in human cancer^3,4^. Phosphatase and tensin homolog (PTEN) functions as a negative regulator of the PI3K/AKT/PTEN pathway; therefore, its loss results in pathway activation that drives tumor growth^5^. Loss of function of *PTEN* is associated with poor prognosis in BC^6,7^ and the ER+ HER2– subtype (Fig. 1) and has been implicated in resistance to endocrine therapy and CDK4/6 and PI3Kα inhibitors^8–12^. The development of effective therapeutic strategies after progression on these agents remains an unmet need in metastatic BC (MBC). To that end, an understanding of both de novo and acquired driver tumor genomic alterations may unlock precision medicine approaches for patients with this disease.

Capivasertib (AZD5363) is an oral, potent, selective, ATP-competitive pan-AKT kinase inhibitor^13–15^. In a multipart Phase I study (ClinicalTrials.gov, NCT01226316), capivasertib monotherapy and, subsequently, combination therapy with fulvestrant was tested in a number of genomically selected expansion cohorts with expected PI3K/AKT/PTEN pathway activation^14^. Here we report the Phase I expansion cohort evaluating capivasertib and fulvestrant in *PTEN*-mutant, ER+ MBC patients. Study objectives were to confirm^14,16^ safety and tolerability and assess preliminary antitumor activity of the combination therapy in this patient population, and to describe exploratory genomic biomarker analyses of collected circulating tumor DNA (ctDNA) and tumor samples.

## Results

### Patient demographics and disease characteristics

In total, 32 patients with *PTEN*-mutant ER+ MBC were enrolled across eight sites in six countries, of whom 31 patients ultimately received treatment with capivasertib in combination with fulvestrant (*n* = 12 fulvestrant naive and *n* = 19 fulvestrant pretreated). Patient demographics and disease characteristics are shown in Table 1 and Supplementary Table 1. Mean age of study participants was 53 years (range 31–70). Most (90%) patients had visceral disease at enrollment and were heavily pretreated, with a median of 7 (range 1–13) prior therapies. Although the median number (7) of prior anticancer regimens was equivalent in the cohorts, fulvestrant-naive patients received more prior lines of chemotherapy (median 4 vs 2) and fewer lines of endocrine therapy (median 2 vs 4) compared with fulvestrant-pretreated patients. The slightly higher rates of visceral involvement (100% vs 84%) and progesterone-receptor-negative status (17% vs 5%) observed in the fulvestrant-naive versus fulvestrant-resistant patients may also support this observation. Patients in the fulvestrant-pretreated group were also more likely to have received prior mTOR inhibitors (48% vs 25%), CDK4/6 inhibitors (63% vs 8%) and PI3K inhibitors (11% vs 0%) than fulvestrant-naive patients (Table 1).

**Table 1:** Demographic and disease characteristics in 31 *PTEN*-mutant^a^ ER+ MBC patients treated with capivasertib and fulvestrant.

Characteristics	Fulvestrant naive (*N* = 12)	Fulvestrant pretreated (*N* = 19)
*Mean age, years (range)*	51 (31–68)	55 (42–70)
*Female gender, n (%)*	12 (100)	19 (100)
*Race, n (%)*		
White	10 (83)	16 (84)
Black or African American	1 (8)	1 (5)
Asian	0	1 (5)
Other	1 (8)	1 (5)
*WHO performance status, n (%)*		
0, normal activity	5 (42)	8 (42)
1, restricted activity	7 (58)	11 (58)
*Visceral disease, n (%)*	12 (100)	16 (84)
*ER+ and PR+, n (%)*^b^	10 (83)	17 (89)^c^
*ER+ and PR–, n (%)*^b^	2 (17)	1 (5)^c^
*HER2+, n (%)*^b^	1 (8)	2 (11)
*Median number of prior anticancer regimens (range)*^d^	7 (1–11)	7 (3–13)
Chemotherapy^e^	4 (0–10)	2 (1–8)
Endocrine therapy^e^	2 (0–4)	4 (1–6)
*Prior CDK4/6 inhibitor, n (%)*	1 (8)	12 (63)
*Prior mTOR inhibitor, n (%)*	3 (25)	9 (47)
*Prior PI3K inhibitor, n (%)*	0	2 (11)

Thirty-two patients were enrolled, but 1 patient deteriorated prior to starting study treatment; therefore, data are presented here for 31 treated patients.

*ER+* estrogen-receptor-positive, *HER2+* human epidermal growth factor receptor positive, *IHC* immunohistochemistry, *MBC* metastatic breast cancer, *PR+/–*, progesterone-receptor-positive/negative.

^a^A *PTEN* alteration was detected in all patients.

^b^Includes both primary and metastatic biopsies.

^c^Progesterone receptor status was missing for one patient in the fulvestrant-pretreated cohort.

^d^Inclusive of adjuvant and metastatic therapies received.

^e^Exploratory analyses.

### Efficacy analyses

**Fig. 2:**
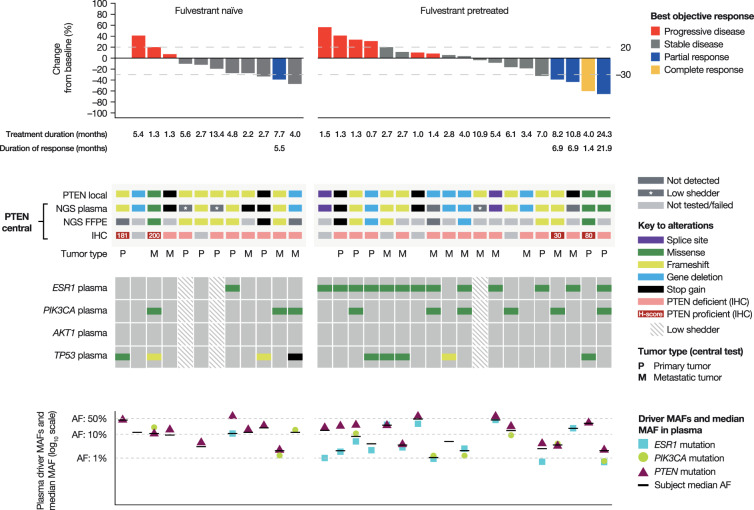
Best RECIST response, *PTEN*, and broader mutation profiling in patients with *PTEN*-mutant ER+ MBC treated with capivasertib and fulvestrant. AF allele fraction, ER+ estrogen-receptor-positive, FFPE formalin-fixed paraffin-embedded, IHC immunohistochemistry, MAF mutant allele fraction, MBC metastatic breast cancer, NGS next-generation sequencing, RECIST Response Evaluation Criteria in Solid Tumors.

At data cut-off (June 2019), three patients remained on study treatment, the majority having discontinued because of disease progression (*n* = 24; Supplementary Fig. 1). Response Evaluation Criteria in Solid Tumors (RECIST) response data are presented in Fig. 2 and summarized by prior fulvestrant exposure in Table 2. Of 30 patients with available RECIST data at baseline and at least one follow-up assessment, 17 (57%) demonstrated target lesion shrinkage (Fig. 2). Objective response rate (ORR) was 8% (1/12, 95% confidence interval [CI] 0–39) in fulvestrant-naive patients and 21% (4/19, 95% CI 6–46) in fulvestrant-pretreated patients; clinical benefit rate at 24 weeks (CBR_24_) was 17% (2/12, 95% CI 2–48) and 42% (8/19, 95% CI 20–67), respectively. Median duration of response (DOR) was 169 days (95% CI not calculable) in the fulvestrant-naive cohort (*n* = 1) and 210 days (95% CI 43–670) in the fulvestrant-pretreated cohort (*n* = 4). Median progression-free survival (PFS) was 2.7 months (95% CI 2–4) in all patients (*n* = 31): fulvestrant naive 2.6 months (95% CI 1–4), fulvestrant pretreated 4.1 months (95% CI 2–7).

**Table 2:** Clinical efficacy summary in patients with *PTEN*-mutant ER+ MBC treated with capivasertib and fulvestrant.

	Fulvestrant naive (*N* = 12)	Fulvestrant pretreated (*N* = 19)	Total (*N* = 31)
Objective response rate, % (95% CI)	8 (0–39)	21 (6–46)	16 (6–34)
Stable disease ≥24 weeks, *n* (%)	1 (8)	4 (21)	5 (16)
Clinical benefit rate, % (95% CI)^a^	17 (2–48)	42 (20–67)	32 (17–51)
Median progression-free survival, months (95% CI)	2.6 (1–4)	4.1 (2–7)	2.7 (2–4)

*CI* confidence interval, *ER+* estrogen-receptor-positive, *MBC* metastatic breast cancer.

^a^Percentage of responders (patients who had a confirmed partial or complete response) plus those with stable disease for ≥24 weeks.

### Safety and therapy exposure

Median treatment duration (at data cut-off) was 103 days (range 22–740) overall: 102.5 days (24–410) in fulvestrant-naive patients and 103 days (22–740) in fulvestrant-pretreated patients. All patients experienced adverse events (AEs; Table 3), the most common of which were diarrhea (*n* = 21, 68%) and nausea (*n* = 14, 45%). Grade ≥3 AEs occurred in 17 patients (55%), most frequently maculopapular rash (*n* = 3, 10%), headache and diarrhea (both *n* = 2, 7%). Twelve grade ≥3 AEs occurring in 10 patients were considered causally related to capivasertib, of which both diarrhea and maculopapular rash were seen in two patients. Serious AEs (Supplementary Table 2) were reported in 11 patients (36%), 3 (10%) of which were considered causally related to capivasertib (malaise, nausea, vomiting). Capivasertib dose reduction and discontinuation was required because of diarrhea (*n* = 1, 3%) and drug hypersensitivity (*n* = 1, 3%), respectively. No treatment-related or AE-attributable deaths in either cohort were observed.

**Table 3:** AEs occurring in >10% of treated patients irrespective of causality.

MedDRA preferred term, *n* (%)	All patients (*n* = 31)	
	All grades	Grade ≥3
Patients with any AE	31 (100)	17 (55)
Diarrhea	21 (68)	2 (6)
Nausea	14 (45)	1 (3)
Headache	9 (29)	2 (6)
Vomiting	9 (29)	1 (3)
Dizziness	7 (23)	0
Fatigue	7 (23)	0
Abdominal pain	5 (16)	1 (3)
Constipation	5 (16)	0
Hyperglycemia	5 (16)	1 (3)
Rash maculopapular	5 (16)	3 (10)
Decreased appetite	4 (13)	0
Flatulence	4 (13)	0
Pruritus	4 (13)	0

*AE* adverse event, *MedDRA* Medical Dictionary for Regulatory Activities.

### Exploratory biomarker analyses

Thirty-one patients were enrolled based on detection of an eligible *PTEN* alteration by local testing of tumor tissue (*n* = 29) or ctDNA (*n* = 2; Fig. 2). The majority of detected *PTEN* alterations led to a premature stop codon (21, 68%; frameshift, *n* = 14; stop gain, *n* = 5; splice, *n* = 2). Actionable missense mutations were detected in three (10%) patients, and a *PTEN* gene deletion was detected in the remaining seven (23%). Although numbers were very small, there was no apparent correlation between the type of *PTEN* alteration and clinical response during the study. Central next-generation sequencing (NGS) of plasma and/or tissue detected the *PTEN* alteration in 28 of the 30 (93%) cases with evaluable samples. Central immunohistochemistry (IHC; *n* = 25) showed complete loss of PTEN protein in 21 cases (84%), with various levels of *PTEN* expression in the remaining four evaluable samples. Overall, evidence of non-functional *PTEN* by central testing was confirmed in all patients by NGS or IHC.

Interestingly, two *PTEN*-positive cases by IHC had a *C124S PTEN* mutation by NGS, a dominant-negative form of *PTEN* reported to inhibit phosphatase activity^17^. Intriguingly, one of these patients had a complete response (CR) to study therapy (Fig. 2). The two other *PTEN*-positive cases may have been related to potential heterogeneous expression of the *PTEN* mutations; for one patient, central NGS analysis of the *PTEN*-positive primary breast tumor did not identify a *PTEN* alteration, whereas the central ctDNA analysis identified the same *PTEN* alteration as detected by local NGS analysis of a liver metastasis. For the other patient, low-intensity PTEN protein expression was identified in only a small subset of tumor cells (30%).

Broader genetic profiling by NGS of baseline ctDNA samples revealed co-occurring alterations in *PIK3CA* in 9/28 (32%) patients with sufficient tumor DNA for analysis (Fig. 2), a slightly greater rate than that (19–20%) reported by The Cancer Genome Atlas and METABRIC analyses^18–20^. Three of the four cases with a partial response (PR) had a co-occurring *PIK3CA* mutation, but the association of co-occurring *PIK3CA* mutations with PFS did not reach statistical significance (*P* = 0.15) in this small cohort. As expected, there was a higher prevalence of co-occurring *ESR1* mutations in the fulvestrant-pretreated compared with the fulvestrant-naive cohort (72% vs 10%, respectively). In contrast, co-occurring *TP53* mutations were more prevalent in the fulvestrant-naive than in the fulvestrant-pretreated cohort (40% vs 28%, respectively). No other mutations in the PI3K pathway were identified. A closer look at the comparison of the *PTEN* mutant allele fractions (MAFs) with the median of the co-occurring mutation MAFs in each patient showed that *PTEN* was detected at or above the median MAF in almost all patients, suggesting *PTEN* as the dominant driver mutation in those patients (Fig. 2).

## Discussion

This Phase I expansion study reported on the safety and efficacy of the pan-AKT inhibitor, capivasertib, in combination with the ER antagonist, fulvestrant, in a genomically selected advanced ER+ BC population harboring an eligible deleterious *PTEN* gene alteration in the tumor. *PTEN* mutations select for an aggressive genomic subtype of ER+ BC, with associated resistance to standard-of-care therapies.

Capivasertib plus fulvestrant had an acceptable safety profile that was consistent with prior data^16^ and demonstrated antitumor activity in this heavily pretreated patient cohort (median 7 prior therapies), including in those previously treated with fulvestrant. Although efficacy appeared marginally better in fulvestrant-pretreated than in fulvestrant-naive patients, there were notable phenotypic and genomic differences between these patient cohorts. Specifically, at enrollment, fulvestrant-naive patients had more visceral disease, received less prior endocrine and more chemotherapy, and, likely reflective of this prior therapy receipt, had a lower *ESR1-* and higher *TP53-*mutation rate, which indeed could also be indicative of a more aggressive disease biology at baseline^21^. Overall, however, and given the poor prognostic genomic subgroup selected for this study, reasonable efficacy (ORR 21%; CBR_24_ 42%) was seen in the fulvestrant-pretreated cohort. On the whole, this clinical dataset supports prior observations^7,22^ suggesting *PTEN* as a negative prognostic biomarker in BC, given the relatively short median PFS (2.7 [95% CI 2–4]) duration observed across the study population.

In this multicenter international study, local testing was reliable, with central retrospective confirmation of non-functional *PTEN* status achieved in all patients. Importantly, *PTEN* did appear to be the dominant driver tumor mutation in this study population. In addition, the trial adds support for enrollment in genomically selected studies to be based primarily on local testing, thus avoiding delays to study accruals from the impact of central testing, particularly in early-phase signal-seeking studies such as these.

The incorporation of mTOR and CDK4/6 inhibitors into endocrine therapy has led to substantial improvements in patient outcomes^23–27^. Almost half of our *PTEN*-mutant study population had received prior CDK4/6 inhibitor therapy. This is of particular interest given recent data proposing *PTEN* inactivation as a mechanism of resistance to this therapeutic class as well as PI3Kα-selective inhibitors, lending support to direct AKT inhibitors in this setting^9,11,28^.

Notably, in the Phase I/II randomized FAKTION study, which demonstrated a PFS benefit with the addition of capivasertib to fulvestrant in a molecularly unselected, aromatase-inhibitor-resistant but fulvestrant-naive ER+ MBC population, a subgroup of patients with *PIK3CA* mutation (by digital droplet polymerase chain reaction) and/or *PTEN* loss (by IHC) did not appear to have any greater sensitivity to the combination than those without the predefined alterations. Importantly, however, no FAKTION participants had received previous CDK4/6 inhibitor therapy, and the rate of *AKT1* and *PTEN* mutations in that study has not yet been reported^29^.

Our study had several important limitations, including the trial not being formally powered to compare efficacy across fulvestrant-naive and -pretreated cohorts, as well as the small patient numbers. It is also noteworthy that the fulvestrant-naive patients enrolled may have had a more aggressive disease phenotype and a poorer prognosis than the fulvestrant-pretreated cohort, although the sample size limited any formal comparisons. At the planned interim analysis, the fulvestrant-naive cohort did not meet its target value for CBR_24_, so recruitment was halted, resulting in a cohort of only 12 patients. Furthermore, the rarity of this biomarker led to slow accrual in the fulvestrant-pretreated cohort (approximately 29 months), with the result that this cohort was closed before reaching the target of 24 patients.

In conclusion, this study shows that capivasertib in combination with fulvestrant has clinical activity in heavily pretreated *PTEN*-mutant ER+ MBC patients, a poor prognostic BC subtype. *PTEN* was the dominant driver tumor mutation in these patients. Further analyses of patients pretreated with a CDK4/6 inhibitor in the ongoing Phase III study CAPItello-291 (NCT04305496), which is evaluating combination capivasertib with fulvestrant, will definitively inform *PTEN’*s role as a therapeutic target in BC. In the Phase I study reported here, this aggressive disease entity appeared to display unique biology with at least a subset dependent on AKT and ER for proliferation, an observation that may benefit therapeutically from using an AKT inhibitor combination.

## Methods

### Study design and participants

This was a Phase I, dose- and schedule-finding study of capivasertib with multiple expansion cohorts that included evaluation of capivasertib with fulvestrant (NCT01226316). Results from the earlier parts of the study presenting the dose-finding, recommended Phase II dose and pharmacodynamic evaluation, as well as efficacy in patients with advanced solid tumors and those with activating *PIK3CA*^30^ or *AKT1* mutations, have previously been reported^14,16^. The final part of this study enrolled *PTEN*-mutant ER+ MBC patients into two subcohorts: fulvestrant naive and fulvestrant pretreated (with a maximum of 24 patients per cohort).

### Inclusion criteria

Eligible patients ≥18 years old had histological or cytological confirmation of ER+ advanced or MBC refractory to standard therapies and confirmation of an eligible *PTEN* alteration in tumor tissue by local testing. Eligible *PTEN* alterations were defined as a deleterious mutation in *PTEN* or copy loss of the *PTEN* gene^31^. Further inclusion criteria were measurable disease by RECIST v1.1, WHO performance status 0–1, and minimum life expectancy of 12 weeks. Key exclusion criteria included active central nervous system metastases, prior treatment with catalytic AKT inhibitors (prior exposure to all other agents in the PI3K/AKT/mTOR pathway, including allosteric AKT inhibitors, was allowed), and clinically significant abnormalities of glucose metabolism [defined by any of the following criteria: i. diagnosis of diabetes mellitus type I or II (irrespective of management); ii. baseline fasting glucose value of ≥7 mmol/L (fasting is defined as no calorific intake for at least 8 h); and iii. glycated hemoglobin >8% (>64 mmol/mol)].

All patients provided written informed consent, the institutional review boards or independent ethics committees of all investigational sites approved the protocol, and the study was performed in accordance with the Declaration of Helsinki, Good Clinical Practice, and the AstraZeneca policy on bioethics^32^.

### Procedures

Patients were treated with oral capivasertib 400 mg twice daily, 4 days on followed by 3 days off, weekly (cycle length: 21 days), and fulvestrant at the labeled dose, in accordance with the previously established recommended combination dose^29^. Antitumor activity was assessed by computed tomography or magnetic resonance imaging (RECIST v1.1) every 6 weeks for 24 weeks, then every 12 weeks. Safety was assessed throughout the study period and until day 28 after discontinuation of study treatment, according to the National Cancer Institute Common Terminology Criteria for Adverse Events v4.0. AEs were coded with the Medical Dictionary for Regulatory Activities v19.1.

For study enrollment, *PTEN* mutation (with known functional or therapeutic significance as described in Carr et al.^33^) status was determined in tissue/plasma by local NGS and involved a variety of assays in accordance with local standard practice without any specific threshold for positivity. *PTEN* status was also centrally evaluated in tumor tissue from either primary or metastatic disease (as indicated in Fig. 2) by FoundationOne^34^ testing, by IHC analyses of PTEN protein expression (using the CST138G6 PTEN antibody assay with H­score ≤10 classified as PTEN protein deficient)^35^, and in ctDNA by using a hybrid capture-based panel covering 600 genes (AZ600) and low-pass whole-genome sequencing for cases with a *PTEN* gene deletion reported by the local test. *PTEN* was considered clonal if the *PTEN* MAF was greater than the median MAF of the remaining mutations identified in the plasma sample with MAF > 1%. These central analyses were conducted retrospectively.

### Outcomes

Safety and tolerability were assessed by continual monitoring of AEs. Efficacy outcomes included: ORR, defined as a confirmed PR or CR; DOR, defined as the time from first objective response to disease progression or death (or censoring if neither outcome is observed); PFS, defined as the time from the first day of treatment to disease progression or death; and CBR_24_, defined as confirmed disease response (PR or CR) or stabilization for ≥24 weeks. Responses were investigator assessed in accordance with RECIST v1.1 and required a confirmatory scan. Exploratory biomarker analyses included mutation analysis of baseline tissue and ctDNA plasma samples (by NGS), along with analysis of PTEN protein expression in baseline tumor tissue (by IHC).

### Statistical analysis

**Fig. 3:**
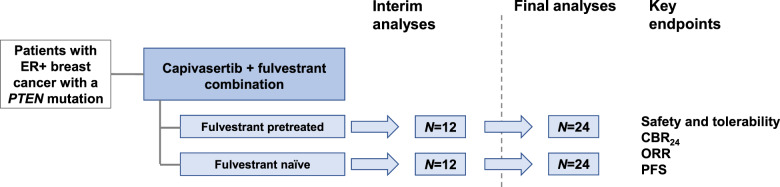
Study design of the *PTEN*-mutant breast cancer cohort. Up to 24 patients in each cohort. Interim analyses were carried out after 12 patients were followed up for 24 weeks or withdrawn from the study. Subsequent patients were recruited only if sufficient antitumor activity (assessed by CBR_24_) was observed at the interim analyses. CBR_24_ clinical benefit rate at 24 weeks, ER+ estrogen-receptor-positive, ORR objective response rate, PFS progression-free survival.

Although the primary endpoint throughout this multipart Phase I study remained safety and tolerability, the sample size of the Phase I expansion cohort reported here was determined with the aim of detecting a signal of efficacy, should one exist, using CBR_24_. The sample size was determined based on prespecified CBR_24_ target values of 65% and 40% for fulvestrant-naive and fulvestrant-pretreated patients, respectively. With 24 patients per cohort (Fig. 3), there would be a 90% chance of at least 13 and 7 clinical benefit responses, respectively. At the planned interim analysis, the fulvestrant-naive cohort did not meet its target (CBR_24_ of 65%), and recruitment was halted. The fulvestrant-pretreated cohort did meet its predefined boundary (CBR_24_ of 40%), and recruitment continued. However, subsequent to this and owing to the difficulty in recruiting this rare patient population, this latter cohort was also closed before the target of 24 patients was reached. It was considered that sufficient data were available from the 19 patients dosed in this fulvestrant-pretreated cohort to allow a reasonable chance of assessing any signal of efficacy. Final analyses were conducted when all patients had the opportunity to reach 24 weeks of treatment for assessment of CBR_24_. DOR and PFS were analyzed by Kaplan–Meier plots, and patients without a progression event at the analysis date were censored at the last known RECIST assessment. Exploratory biomarker analyses investigated the association between mutations and radiographic response. All analyses were conducted with SAS v9.04.

### Reporting summary

Further information on research design is available in the Nature Research Reporting Summary linked to this article.

## Supplementary information

Supplementary material

Reporting Summary

## Data Availability

The raw sequencing data are not publicly available because of data privacy regulations and restrictions for use of such data, as stated in the study protocol and patient consent form. Individual-level data can potentially be accessed via a collaborative agreement with AstraZeneca Group. The authors declare that the clinical dataset analyzed here, including PFS and tumor response data, is available and may be obtained in accordance with AstraZeneca’s data sharing policy as part of an external collaborative request (https://astrazenecagroup-dt.pharmacm.com//DT/Home/Index/) or an external data access request (https://vivli.org/ourmember/astrazeneca/). The data generated and analyzed during this study are described in the following metadata record: 10.6084/m9.figshare.14192345^36^.
