# Supplementary material for: Selective AKT kinase inhibitor capivasertib in combination with fulvestrant in PTEN-mutant ER-positive metastatic breast cancer
Source: NPJ Breast Cancer. 2021 Apr 16;7:44. doi: 10.1038/s41523-021-00251-7 (PMC8052445; doi:10.1038/s41523-021-00251-7)
Supplement: Supplementary file 1 — Supplementary material [file 41523_2021_251_MOESM1_ESM.pdf]

Selective AKT kinase inhibitor capivasertib in combination with fulvestrant in *PTEN*-mutant ER-positive metastatic breast cancer

Supplementary material

Supplementary Table 1. Estrogen and progesterone receptor status by site of disease

No. of patients	Site of disease		
(N=31)	**Primary**	**Metastatic**	**Unknown**
15	–	ER+ and PR+	–
10	ER+ and PR+	–	–
3	–	ER+ and PR-	–
1	PR+	ER+	–
1	–	ER+	PR unknown
1	–	–	ER+ and PR+

ER+, estrogen receptor positive; PR+, progesterone receptor positive

Supplementary Table 2. Number of patients experiencing AEs, and dose modifications and discontinuations due to AEs and serious AEs, during treatment with capivasertib and fulvestrant

***n* (%)**	**Fulvestrant naïve (*N*=12)**	**Fulvestrant pretreated (*N*=19)**	**Total (*N*=31)**
AE of any grade causally related to capivasertib	11 (91.7)	18 (94.7)	29 (93.5)
Any AE of grade ≥3	6 (50.0)	11 (57.9)	17 (54.8)
Any AE leading to death	0	0	0
Any AE leading to discontinuation of capivasertib	0	1 (5.3)[1]	1 (3.2)
Any AE leading to dose reduction of capivasertib	0	1 (5.3)[2]	1 (3.2)
Any serious AE	6 (50.0)	5 (26.3)	11 (35.5)
Any causally related serious AE	1 (8.3)	2 (10.5)	3 (9.7)

[1]Drug hypersensitivity; [2]Diarrhea. AE, adverse event

Supplementary Figure 1. Study consort diagram

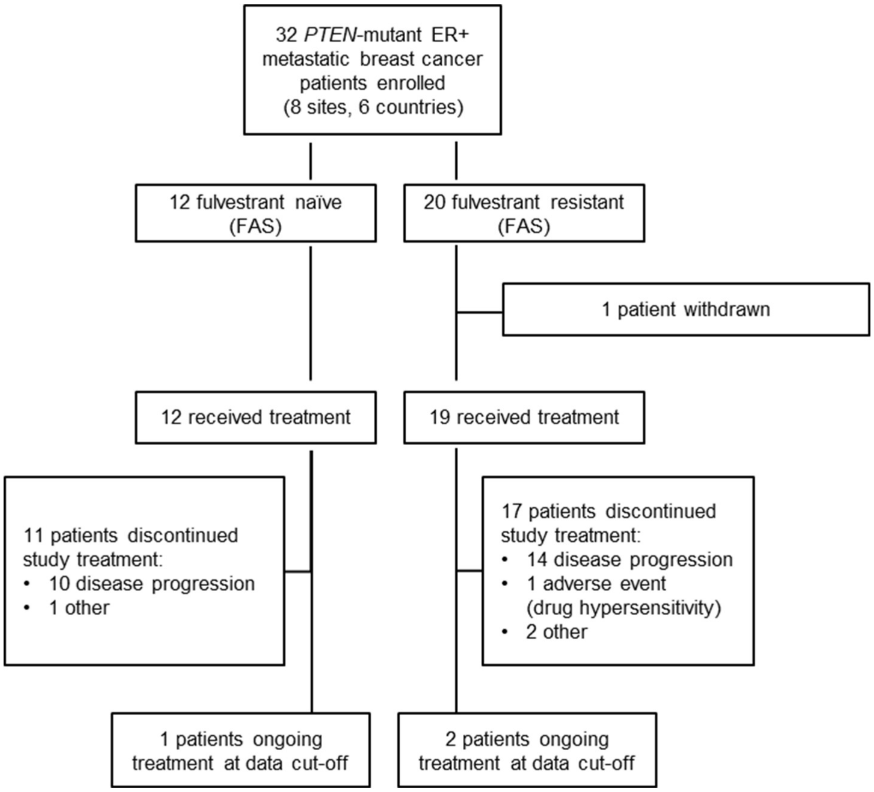

ER+, estrogen receptor positive; FAS, full analysis set
