# Supplementary material for: Selective AKT kinase inhibitor capivasertib in combination with fulvestrant in PTEN-mutant ER-positive metastatic breast cancer
Source: NPJ Breast Cancer. 2021 Apr 16;7:44. doi: 10.1038/s41523-021-00251-7 (PMC8052445; doi:10.1038/s41523-021-00251-7)
Supplement: Supplementary file 2 — Reporting Summary [file 41523_2021_251_MOESM2_ESM.pdf]

nature research

Corresponding author(s): Sarat Chandarlapaty

Last updated by author(s): Jan 27, 2021

Reporting Summary

Nature Research wishes to improve the reproducibility of the work that we publish. This form provides structure for consistency and transparency in reporting. For further information on Nature Research policies, see our Editorial Policies and the Editorial Policy Checklist.

Statistics

For all statistical analyses, confirm that the following items are present in the figure legend, table legend, main text, or Methods section.

n/a	Confirmed	
☐	☒	The exact sample size (n) for each experimental group/condition, given as a discrete number and unit of measurement
☒	☐	A statement on whether measurements were taken from distinct samples or whether the same sample was measured repeatedly
☐	☒	The statistical test(s) used AND whether they are one- or two-sided<br>*Only common tests should be described solely by name; describe more complex techniques in the Methods section.*
☒	☐	A description of all covariates tested
☐	☒	A description of any assumptions or corrections, such as tests of normality and adjustment for multiple comparisons
☐	☒	A full description of the statistical parameters including central tendency (e.g. means) or other basic estimates (e.g. regression coefficient) AND variation (e.g. standard deviation) or associated estimates of uncertainty (e.g. confidence intervals)
☒	☐	For null hypothesis testing, the test statistic (e.g. F, t, r) with confidence intervals, effect sizes, degrees of freedom and P value noted<br>*Give P values as exact values whenever suitable.*
☒	☐	For Bayesian analysis, information on the choice of priors and Markov chain Monte Carlo settings
☒	☐	For hierarchical and complex designs, identification of the appropriate level for tests and full reporting of outcomes
☒	☐	Estimates of effect sizes (e.g. Cohen's d, Pearson's r), indicating how they were calculated

Our web collection on statistics for biologists contains articles on many of the points above.

Software and code

Policy information about availability of computer code

Data collection	Web Based Data Capture was used for data collection and query handling
Data analysis	SAS v9.04.

For manuscripts utilizing custom algorithms or software that are central to the research but not yet described in published literature, software must be made available to editors and reviewers. We strongly encourage code deposition in a community repository (e.g. GitHub). See the Nature Research guidelines for submitting code & software for further information.

Data

Policy information about availability of data

All manuscripts must include a data availability statement. This statement should provide the following information, where applicable:

- Accession codes, unique identifiers, or web links for publicly available datasets
- A list of figures that have associated raw data
- A description of any restrictions on data availability

Data underlying the findings described in this manuscript may be obtained in accordance with AstraZeneca's data-sharing policy described at: https://astrazenecagrouptrials.pharmacm.com/ST/Submission/Disclosure.

Field-specific reporting

Please select the one below that is the best fit for your research. If you are not sure, read the appropriate sections before making your selection.

☒ Life sciences ☐ Behavioural & social sciences ☐ Ecological, evolutionary & environmental sciences

For a reference copy of the document with all sections, see nature.com/documents/nr-reporting-summary-flat.pdf

Life sciences study design

All studies must disclose on these points even when the disclosure is negative.

Sample size	The sample size was determined based on prespecified clinical benefit rate at 24 weeks target values of 65% and 40% for fulvestrant-naïve and fulvestrant-pretreated patients, respectively. With 24 patients per cohort, there would be a 90% chance of at least 13 and seven clinical benefit responses, respectively
Data exclusions	No data were excluded
Replication	Not applicable because we report an open-label clinical study
Randomization	No randomisation was performed because we report an open-label clinical study
Blinding	No blinding was performed because we report an open-label clinical study

Reporting for specific materials, systems and methods

We require information from authors about some types of materials, experimental systems and methods used in many studies. Here, indicate whether each material, system or method listed is relevant to your study. If you are not sure if a list item applies to your research, read the appropriate section before selecting a response.

Materials & experimental systems

n/a	Involved in the study	
☒	☐	Antibodies
☒	☐	Eukaryotic cell lines
☒	☐	Palaeontology and archaeology
☒	☐	Animals and other organisms
☐	☒	Human research participants
☐	☒	Clinical data
☒	☐	Dual use research of concern

Methods

n/a	Involved in the study	
☒	☐	ChIP-seq
☒	☐	Flow cytometry
☒	☐	MRI-based neuroimaging

Human research participants

Policy information about studies involving human research participants

Population characteristics	Characteristics of the patients, including medical history and disease characteristics at baseline were collected. For example, age, gender, race, WHO performance status, visceral disease, estrogen and progesterone receptor status, HER2 status, prior anticancer regimens, prior CDK4/6 inhibitors, prior mTOR inhibitors, prior PI3K inhibitors, and smoking history
Recruitment	Upon screening patients' tumours for mutations, patients with advanced or metastatic ER+ breast cancer that is positive for selected PTEN mutations were recruited. This includes two cohorts of patients: patients with prior fulvestrant treatment and those without
Ethics oversight	The study was performed in accordance with ethical principles that have their origin in the Declaration of Helsinki and are consistent with the International Conference on Harmonisation (ICH)/Good Clinical Practice (GCP) guidelines, applicable regulatory requirements and the AstraZeneca policy on Bioethics and Human Biological Samples.

Note that full information on the approval of the study protocol must also be provided in the manuscript.

Clinical data

Policy information about clinical studies

All manuscripts should comply with the ICMJE guidelines for publication of clinical research and a completed CONSORT checklist must be included with all submissions.

Clinical trial registration	NCT01226316

Study protocol	Submitted with the manuscript
Data collection	Patients were enrolled across eight recruitment sites in six countries
Outcomes	Safety and tolerability were assessed by continual monitoring of AEs. Efficacy outcomes included: ORR, defined as a confirmed partial response (PR) or complete response (CR); DOR, defined as the time from first objective response to disease progression or death (or censoring if neither outcome is observed); PFS, defined as the time from the first day of treatment to disease progression or death; and CBR24, defined as confirmed disease response (PR or CR) or stabilization for ≥24 weeks. Responses were investigator assessed in accordance with RECIST v1.1 and required a confirmatory scan. Exploratory biomarker analyses included mutation analysis of baseline tissue and ctDNA plasma samples (by NGS), along with analysis of PTEN protein expression in baseline tumor tissue (by IHC).
